# Hyperacusis: major research questions

**DOI:** 10.1007/s00106-017-0464-3

**Published:** 2018-02-01

**Authors:** D. M. Baguley, D. J. Hoare

**Affiliations:** 1 NIHR Nottingham Biomedical Research Centre, Ropewalk House, 113 The Ropewalk, NG1 5DU Nottingham, UK; 2 Otology and Hearing Group, Division of Clinical Neuroscience, School of Medicine, University of Nottingham, Nottingham, UK

**Keywords:** Hyperacusis, Sound tolerance, Tinnitus, Audiology, Pathology, Hyperakusis, Geräuschtoleranz, Tinnitus, Audiologie, Pathologie

## Abstract

**Background:**

Hyperacusis is a troublesome symptom that can have a marked negative impact on quality of life.

**Objectives:**

To identify major research questions in hyperacusis.

**Materials and methods:**

Review of gaps in knowledge regarding hyperacusis, and where opportunities may lie to address these.

**Results:**

Eight major research questions were identified as priorities for future research. These were: What is the prevalence of hyperacusis in adults and children? What are the risk factors associated with hyperacusis? What is the natural history of hyperacusis? How is ‘pain hyperacusis’ perceived? What mechanisms are involved in hyperacusis? What is the relationship between hyperacusis and tinnitus? Can a questionnaire be developed that accurately measures the impact of hyperacusis and can be used as a treatment outcome measure? What treatments, alone or in combination, are effective for hyperacusis?

**Conclusion:**

This clinical/researcher-led project identified major research questions in hyperacusis. A further development to identify patient-prioritized research will follow.

## Zusammenfassung

**Hintergrund:**

Geräuschempfindlichkeit oder Hyperakusis stellt ein belastendes Symptom dar, das einen ausgeprägten negativen Einfluss auf die Lebensqualität haben kann.

**Ziel:**

Ziel der vorliegenden Arbeit war es, zentrale Fragestellungen der Forschung zur Hyperakusis zu ermitteln.

**Material und Methoden:**

Dazu wird ein Überblick über bisher existierende Lücken im Wissen zur Hyperakusis gegeben sowie mögliche Ansätze der weiteren Erforschung dargestellt.

**Ergebnisse:**

Es wurden 8 zentrale Fragestellungen ermittelt, deren Beantwortung Priorität für die zukünftige Forschung haben sollte: Wie hoch ist die Prävalenz der Hyperakusis bei Erwachsenen und Kindern? Welches sind die mit Hyperakusis verbundenen Risikofaktoren? Wie ist der natürliche Verlauf der Hyperakusis? Wie wird Schmerz infolge Hyperakusis wahrgenommen? Welche Mechanismen tragen zur Entstehung der Hyperakusis bei? Wie ist der Zusammenhang zwischen Hyperakusis und Tinnitus? Kann ein Fragebogen entwickelt werden, der den Einfluss der Hyperakusis genau misst, und kann er als Ergebnisparameter für die Beurteilung der Behandlung eingesetzt werden? Welche Therapien, allein oder in Kombination, sind bei der Hyperakusis wirksam?

**Schlussfolgerung:**

Im Rahmen des vorliegenden sowohl von Klinikern als auch Wissenschaftlern geleiteten Projekts wurden zentrale Fragestellungen der Forschung zur Hyperakusis ermittelt. Es wird eine Weiterentwicklung der Frage dahingehend folgen, welche Forschungsansätze Patienten priorisieren.

## Background

The term “hyperacusis” is used to describe the experience of everyday sounds being perceived as intense and overwhelming. Other terminology that is used in this regard includes “decreased” or “reduced sound tolerance”: An Internet patient forum (www.hyperacusis.net [23]) uses the variant “collapsed sound tolerance.” While there is undoubtedly an emotional and psychological component to hyperacusis [25] (not least since becoming apprehensive about sound exposure is an obvious corollary to perceiving that sound as intense), hyperacusis is a subjective self-reported symptom of some physiological change in the central auditory system such as increased gain [3], such that even when sound is of a moderate intensity it is perceived as loud and intrusive. Hyperacusis is almost exclusively bilateral, and the presentation of unilateral hyperacusis is confined to unilateral triggers such as an acoustic shock [30] or a specific unilateral neural lesion [7].

Hyperacusis is almost exclusively bilateral

**Fig. 1:**
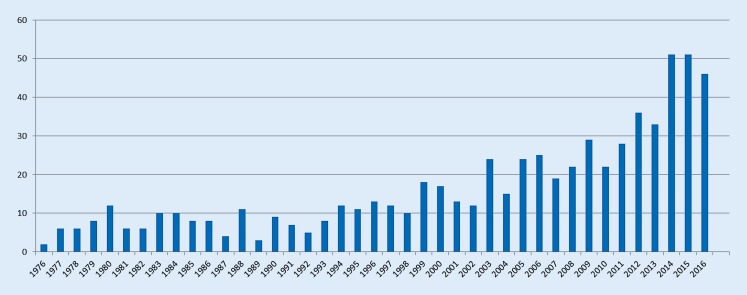
Papers with hyperacusis as a major topic by year (adapted from [5])

Interest in hyperacusis from both clinicians and researchers is gathering pace, and the numbers of peer-reviewed scientific papers published on the topic of hyperacusis in the past four decades has increased on an annual basis (Fig. 1). Despite this burgeoning attention to symptoms of decreased sound tolerance, fundamental questions remain. The purpose of the present article is to describe and delineate several of these questions, with the aim of supporting research efforts to gather evidence on hyperacusis.

## Epidemiology and natural history

With a subjective symptom such as hyperacusis, estimates of the prevalence in the general population will be strongly influenced by how the question about the experience is formulated. Variation in such questions makes comparison across studies challenging, and a recent systematic review [33] considering hyperacusis in childhood and adolescence concluded that such comparison was not possible at present. While it is not possible to generalize across studies of childhood hyperacusis, some data are available. Hall and colleagues [17] reported an epidemiological study in the UK, wherein children aged 11 years were asked about over-sensitivity or distress to particular sounds in a wider survey of hearing and tinnitus. Of the 7096 children involved, 3.7% responded affirmatively to being asked whether they, “ever experience over-sensitivity or distress to particular sounds?” This equates to one child in every typical UK classroom (about 30 children). Risk factors included male gender, higher maternal education level, and readmission to hospital in the first 4 weeks of life.

The situation is much the same regarding the prevalence of hyperacusis in adults, and some basic information about the epidemiology of hyperacusis in adults is not yet available. Paulin and colleagues [34] investigated hyperacusis in a substudy of the Västerbotten Environmental Health Study in Sweden. Of 8520 adults contacted from the general population, 3406 (40.6%) consented to participation in the study, and it is possible that hyperacusis is over-represented as a result of the low response rate. Of the responders, 9.2% self-identified as having hyperacusis, saying “yes” to: “Do you have a hard time tolerating everyday sounds that you believe most other people can tolerate?”; 1.9% had been diagnosed with sound intolerance by a physician (there was unspecified overlap between the groups). The length of history was not reported.

For some people, hyperacusis is a long-term condition

A question that often arises when counseling a patient with hyperacusis is that of the natural history of the condition. As with epidemiology, basic information is not yet available in this regard, and presently it is not possible to be certain about the future trajectory of a person with hyperacusis. It is evident from patient forums that for some people hyperacusis is a long-term condition, and that for some it is marked by exacerbation because of repeated exposure to intense environmental sound such as a vehicle horn or an alarm. Since it is likely that other persons in whom the hyperacusis resolved would not be posting on a hyperacusis forum, the possible existence of such individuals would not be apparent. This gap in knowledge could be resolved by a longitudinal population study of persons (adults and children) self-reporting with hyperacusis, with the aim of determining their progress (or otherwise) over time, or by the synthesis of no-intervention control groups in clinical trials on hyperacusis (for an example in tinnitus, see Phillips et al. [35]).

Tyler and colleagues [41] have proposed a framework for categorizing patients with hyperacusis on the basis of the defining feature of their experience, suggesting loudness, annoyance, fear, and pain as the important characteristics. While in clinical practice it may not be easy to disambiguate these categories, drawing attention to the experience of sound-evoked pain is of interest. Recent physiology research [15] has identified a population of fibers in the cochlear nerve that appear to be involved in pain perception, perhaps as a warning of cochlear injury. The possibility that these type II unmyelinated fibers are involved in hyperacusis is a potentially important topic for research.

## Mechanisms

Although there is a consensus building that hyperacusis is underpinned by an aberrant increase in central auditory gain [4, 29, 44] (whereby “neural activity from more central auditory structures is paradoxically increased at suprathreshold intensities” −4, p1), further and more detailed information is not yet available. In part this is due to the lack of a satisfactory animal model of hyperacusis [12], but it is also the case that several aspects of mechanisms of loudness perception remain obscure [14]. Moreover, the terminology used by the auditory neuroscience community regarding decreased sound tolerance is variable and nonspecific (Table 1).

**Table 1:** Terminology in use regarding mechanisms of hyperacusis in the auditory neuroscience literature

Hyperresponsiveness [37, 38]
Disruption of central auditory system gain [38]
Pathological increased response gain [29]
Central gain enhancement [4]
Neural amplification [4]
Increased nonlinear gain [44]
Heightened responsiveness to sound [36]
Hypervigilance [37]
Central auditory excitability [21]
Hyperexcitability [2]
Central inhibitory deficit [42]
Central sensitization [40]

One potential way forward would be for the auditory neuroscience community to reach a consensus on the terminology and definitions regarding hyperacusis, and then to undertake specific projects detailing how the increased central auditory gain originates, and then persists.

## Association with tinnitus

Common mechanisms of hyperacusis and tinnitus have been proposed [23] because they commonly occur together (Table 2).

**Table 2:** Reports of hyperacusis in patients with a primary complaint of tinnitus

Authors (date)	Number of patients with tinnitus	Percentage of patients with hyperacusis (%)	Notes
Dauman and Bouscou-Faure (2005) [10]	249	79	Participants in measurement questionnaire research
Hiller and Goebel (2006) [22]	4993	7.3	–
Yang et al. (2013) [43]	207	8.7	Increased prevalence of hyperacusis in bilateral vs. unilateral tinnitus but did not reach statistical significance
Scheckleman et al. (2015) [37]	2333	40	Recalculated to include nonresponders
Degeest et al. (2016) [11]	81	22	“Subjective noise tolerance”= usual or always

While there are several studies detailing hyperacusis in persons with a primary complaint of tinnitus, there is less information about tinnitus in persons with a primary complaint of hyperacusis. Anari and colleagues [3] studied 100 adult patients with a primary complaint of hyperacusis, finding that 86% experienced tinnitus, although the severity and impact of tinnitus were not reported.

What is also missing from the literature is information regarding the severity of hyperacusis in a person with a primary complaint of tinnitus, and vice versa. This would be useful when designing interventions that either have to address both symptoms if severe, or focusing on one or other, with a secondary and less severe symptom not requiring direct intervention.

Tinnitus and hyperacusis can be exacerbated by anxiety and stress

Some aspects of the experiences of people with tinnitus, hyperacusis, or both, are convergent. Both tinnitus and hyperacusis can be exacerbated by anxiety and stress, and in each there is an increased incidence of depression. Treatments for each symptom are emerging that utilize elements of cognitive behavioral therapy (CBT) [8, 26], and these can be combined with sound-based therapy.

There are also several aspects of tinnitus and hyperacusis that are markedly divergent, however. Some of these are illustrated in Table 3. This provides further opportunities for clinical research. The areas of divergence are sufficient for one to consider that hyperacusis and tinnitus are quite distinct phenomena, and while both may involve maladaptive change in the central auditory system, the specific mechanisms and manifestations of these changes may be separate, although they may occur together.

**Table 3:** Divergent characteristics of tinnitus and hyperacusis

Tinnitus	Hyperacusis
Often unilateral, or highly lateralized	Almost exclusively bilateral
Somatic modulation is common	Somatic modulation is rare
Often intermittent	Rarely intermittent
Percept can be formless or primitive	Percept is vivid and salient
Self-help can be very effective	Impact of self-help unknown, may be very limited

## How to measure hyperacusis

Several methods exist that attempt to measure hyperacusis. There are techniques for the determination of the loudest sound an individual can tolerate, or is comfortable with, and these include loudness discomfort levels and loudness scaling techniques [1, 31]. The limitations of such procedures are substantial, however, with marked interobserver and test–retest variability [39]. The use of pure-tone stimuli rather than the environmental sounds involved in the lived experience of a person with hyperacusis also limits how generalizable the measure is to real-world difficulties. Unless performed with great care, exposing an individual to sounds at or close to an intensity that evokes discomfort and pain can be unpleasant, and this has the potential to undermine therapeutic rapport. In general, the clinician is advised to proceed with caution regarding such testing.

There are also several questionnaire instruments available to assess hyperacusis, and these are summarized in Table 4. There are concerns regarding each of these. The Geräuschüberempfindlichkeit (GÜF; [32]) was developed as a brief tool to inform treatment needs and planning. This questionnaire is now available in English [6] but the translated version has not been validated.

**Table 4:** Instruments to measure the impact of hyperacusis

Name	Authors (date)	Format	Validation population	Languages available
Geräuschüberempfindlichkeit (GÜF)	Nelting et al. (2002) [32]	27-item self-report	*N* = 226 with hyperacusis	German, English (Blasing et al., 2010) [6]
Hyperacusis Questionnaire (HQ)	Khalfa et al. (2002) [28]	12-item self-report	*N* = 201 general adult population	French, English
Multiple Activity Scale for Hyperacusis (MASH)	Dauman and Bouscau-Faure (2005) [10]	15-item clinician-led questionnaire	*N* = 249 adults with tinnitus (79% also had hyperacusis)	English

The Hyperacusis Questionnaire (HQ; [28]) was developed to characterize and measure hypersensitivity to sound and is the most commonly used measure. However, it has thus far only been validated in the general population, and not in a (clinical) hyperacusis complaint population. Fackrell and colleagues [13] analyzed HQ data from a tinnitus research volunteer population, and proposed a 10-item, two-factor modification of the HQ for measuring hypersensitivity to sound in a tinnitus population. This modified version is yet to be validated in a new tinnitus participant cohort.

The Multiple-Activity Scale for Hyperacusis (MASH; [10]) was developed to assess in which life situations a person is limited by hyperacusis, how annoyed they are by it, how much speech understanding is affected, and how severe it is at different times. It was validated in a tinnitus rather than a hyperacusis population. It does allow a “real-world” impact to be assessed, in that the individual is asked to rate the impact of hyperacusis on the ability to participate in everyday activities. While some of those activities are culture specific, such as attending the cinema or eating at a restaurant, the responder is encouraged to substitute activities when the stated one is not suitable for them. All the available instruments are designed for adults, and would not be appropriate for use with children or adolescents. Given the prevalence of hyperacusis in young people, this is a topic for potentially fruitful research.

## Treatment

There are many unanswered questions about the efficacy of presently available treatments for hyperacusis, and what might constitute an optimal treatment. The use of sound therapy is widespread, and there are two general approaches, both utilizing wide-band noise. The first is to introduce the sound at a quiet and unchallenging level, and then to gradually increase the intensity over a matter of weeks, with the suggestion that this is similar to a graduated exposure program that might be used for desensitization [25]. Alternatively, one might introduce the sound at a quiet and comfortable level and maintain that intensity, the proposal being that the gain of the auditory system is somehow “recalibrated” by that signal. While there are patient self-help reports indicating that pink noise, for example, may be more beneficial than white noise [24], randomized controlled trials (RCT) of these and other sound-based approaches are not yet available.

Another approach used for hyperacusis treatment is CBT. An RCT for CBT in hyperacusis indicated benefit and improvement in measures of sound tolerance [27]. In the case of tinnitus, combining sound-based therapy with elements of CBT has been demonstrated to be beneficial [8], and on the face of it, such combination therapy might also be effective for hyperacusis.

In the case of sound-evoked otalgia, in which pain-sensitive pathways in the cochlear nerve have been implicated, some form of analgesia might be effective. Intratympanic lidocaine has been trialed for tinnitus [9], but the benefits were minimal and the acute side effect of violent vertigo was said to be debilitating. Any effect on hyperacusis, or sound-evoked otalgia, has not been reported.

## Outlook

In this paper we have described several areas where important information is lacking regarding hyperacusis (summary in Table 5). Clinicians and researchers are encouraged to collaborate and undertake work in this area, with the aim of increasing knowledge and ultimately improving the care of patients who experience hyperacusis. Such collaborative and sustained effort is proving of benefit in the adjacent field of tinnitus [16, 18–20].

**Table 5:** Major research questions in hyperacusis

What is the prevalence of hyperacusis in adults and children?
What are the risk factors associated with hyperacusis?
What is the natural history of hyperacusis?
How is “pain hyperacusis” perceived?
What mechanisms are involved in hyperacusis?
What is the relationship between hyperacusis and tinnitus?
Can a questionnaire be developed that accurately measures the impact of hyperacusis and can be used as a treatment outcome measure?
What treatments, alone or in combination, are effective for hyperacusis?

In the case of tinnitus, and more recently mild-to-moderate hearing loss, listening to another voice has also been of benefit; structured and intentional work to listen to the research questions and priorities of patients has helped influence and provide form to the research agenda [18, 20]. Such work is imminent in the field of hyperacusis, and will provide a priority set of research questions that are immediately important to patients and clinicians. In medical research terms, the field of hyperacusis is young and there is a need for capacity building in this challenging yet fascinating area.

## Practical conclusion

- Hyperacusis can have a marked negative impact on quality of life.
- There are still several areas where important information is lacking regarding hyperacusis.
- Clinicians and researchers are encouraged to collaborate so as to increase knowledge and ultimately improve the care of patients with hyperacusis.
- The field of hyperacusis is young and there is a need for capacity building in this challenging yet fascinating area.

## References

[1] Al-Salim SC, Kopun JG, Neely ST, Jesteadt W, Stiegemann B, Gorga MP (2010). Reliability of categorical loudness scaling and its relation to threshold. Ear Hear.

[2] Alkharabsheh A, Xiong F, Xiong B, Manohar S, Chen G, Salvi R, Sun W (2017). Early age noise exposure increases loudness perception – a novel animal model of hyperacusis. Hear Res.

[3] Anari M, Axelsson A, Eliasson A, Magnusson L (1999). Hypersensitivity to sound–questionnaire data, audiometry and classification. Scand Audiol.

[4] Auerbach BD, Rodrigues PV, Salvi RJ (2014). Central gain control in tinnitus and hyperacusis. Front Neurol.

[5] Baguley DM, Baguley DM, Wray N (2017). Hyperacusis and misophonia. British tinnitus annual tinnitus research review.

[6] Bläsing L, Goebel G, Flötzinger U, Berthold A, Kröner-Herwig B (2010). Hypersensitivity to sound in tinnitus patients: an analysis of a construct based on questionnaire and audiological data. Int J Audiol.

[7] Boucher O, Turgeon C, Champoux S, Ménard L, Rouleau I, Lassonde M, Lepore F, Nguyen DK (2015). Hyperacusis following unilateral damage to the insular cortex: a three-case report. Brain Res.

[8] Cima RF, Maes IH, Joore MA, Scheyen DJ, El Refaie A, Baguley DM, Anteunis LJ, van Breukelen GJ, Vlaeyen JW (2012). Specialised treatment based on cognitive behaviour therapy versus usual care for tinnitus: a randomised controlled trial. Lancet.

[9] Coles RR, Thompson AC, O’Donoghue GM (1992). Intra-tympanic injections in the treatment of tinnitus. Clin Otolaryngol Allied Sci.

[10] Dauman R, Bouscau-Faure F (2005). Assessment and amelioration of hyperacusis in tinnitus patients. Acta Otolaryngol..

[11] Degeest S, Corthals P, Dhooge I, Keppler H (2016). The impact of tinnitus characteristics and associated variables on tinnitus-related handicap. J Laryngol Otol.

[12] Eggermont JJ (2018) Animal models of hyperacusis. In Fagelson MF, Baguley DM (eds) Hyperacusis: clinical and research prespectives. Plural, San Diego. (in press)

[13] Fackrell K, Fearnley C, Hoare DJ, Sereda M (2015). Hyperacusis questionnaire as a tool for measuring hypersensitivity to sound in a tinnitus research population. Biomed Res Int.

[14] Florentine M, Florentine M, Popper AN, Fay RR (2011). Loudness. Loudness.

[15] Flores EN, Duggan A, Madathany T, Hogan AK, Márquez FG, Kumar G, Seal RP, Edwards RH, Liberman MC, García-Añoveros J (2015). A non-canonical pathway from cochlea to brain signals tissue-damaging noise. Curr Biol.

[16] Fuller TE, Haider HF, Kikidis D, Lapira A, Mazurek B, Norena A, Brueggemann PG (2017). Different teams, same conclusions? A systematic review of existing clinical guidelines for the assessment and treatment of tinnitus in adults. Front Psychol.

[17] Hall AJ, Humphriss R, Baguley DM, Parker M, Steer CD (2016). Prevalence and risk factors for reduced sound tolerance (hyperacusis) in children. Int J Audiol.

[18] Hall D, Mohamad N, Firkins L, Fenton M, Stockdale D (2013). Identifying and prioritizing unmet research questions for people with tinnitus: the James Lind Alliance Tinnitus Priority Setting Partnership. Clin Investig (Lond).

[19] Hall DA, Haider H, Szczepek AJ, Lau P, Rabau S, Jones-Diette J, Londero A, Edvall NK, Cederroth CR, Mielczarek M, Fuller T, Batuecas-Caletrio A, Brueggemen P, Thompson DM, Norena A, Cima RF, Mehta RL, Mazurek B (2016). Systematic review of outcome domains and instruments used in clinical trials of tinnitus treatments in adults. Trials.

[20] Henshaw H, Sharkey L, Crowe D, Ferguson M (2015). Research priorities for mild-to-moderate hearing loss in adults. Lancet.

[21] Hickox AE, Liberman MC (2014). Is noise-induced cochlear neuropathy key to the generation of hyperacusis or tinnitus?. J Neurophysiol.

[22] Hiller W, Goebel G (2006). Factors influencing tinnitus loudness and annoyance. Arch Otolaryngol Head Neck Surg.

[23] http://hyperacusisresearch.org. Accessed 30 May 2017

[24] http://www.hyperacusis.net. Accessed 30 May 2017

[25] Jastreboff PJ, Hazell JWP (2004). Tinnitus retraining therapy.

[26] Jüris L, Andersson G, Larsen HC, Ekselius L (2014). Cognitive behaviour therapy for hyperacusis: a randomized controlled trial. Behav Res Ther.

[27] Jüris L, Andersson G, Larsen HC, Ekselius L (2013). Psychiatric comorbidity and personality traits in patients with hyperacusis. Int J Audiol.

[28] Khalfa S, Dubal S, Veuillet E, Perez-Diaz F, Jouvent R, Collet L (2002). Psychometric normalization of a hyperacusis questionnaire. ORL J. Otorhinolaryngol. Relat. Spec..

[29] Knipper M, Van Dijk P, Nunes I, Rüttiger L, Zimmermann U (2013). Advances in the neurobiology of hearing disorders: recent developments regarding the basis of tinnitus and hyperacusis. Prog Neurobiol.

[30] McFerran DJ, Baguley DM (2007). Acoustic shock. J Laryngol Otol.

[31] Morgan DE, Wilson RH, Dirks DD (1974). Loudness discomfort level: selected methods and stimuli. J Acoust Soc Am.

[32] Nelting M, Rienhoff NK, Hesse G, Lamparter U (2002). The assessment of subjective distress related to hyperacusis with a self-rating questionnaire on hypersensitivity to sound. Laryngorhinootologie.

[33] Nemholt Rosing S, Hvass Schmidt J, Wedderkopp N, Baguley DM (2016). Prevalence of tinnitus and hyperacusis in children and adolescents: a systematic review. BMJ Open.

[34] Paulin J, Andersson L, Nordin S (2016). Characteristics of hyperacusis in the general population. Noise Health.

[35] Phillips J, McFerran D, Hall D, Hoare DJ (2017). The natural history of tinnitus: a systematic review and meta-analysis of controlled trials. Laryngoscope.

[36] Salloum RH, Yurosko C, Santiago L, Sandridge SA, Kaltenbach JA (2014). (2014) Induction of enhanced acoustic startle response by noise exposure: dependence on exposure conditions and testing parameters and possible relevance to hyperacusis. PLoS ONE.

[37] Scheckleman M, Landgrebe M, Langguth B, TRI Database Study Group (2015). Phenotypic characteristics of hyperacusis in tinnitus. PLoS ONE.

[38] Song JJ, De Ridder D, Weisz N, Schlee W, Van de Heyning Vanneste PS (2014). Hyperacusis-associated pathological resting-state brain oscillations in the tinnitus brain: a hyperresponsiveness network with paradoxically inactive auditory cortex. Brain Struct Funct.

[39] Stephens SD, Blegvad B, Krogh HJ (1977). The value of some suprathreshold auditory measures. Scand Audiol.

[40] Suhnan AP, Finch PM, Drummond PD (2017). Hyperacusis in chronic pain: neural interactions between the auditory and nociceptive systems. Int J Audiol.

[41] Tyler RS, Pienkowski M, Roncancio ER, Jun HJ, Brozoski T, Dauman N, Dauman N, Andersson G, Keiner AJ, Cacace AT, Martin N, Moore BC (2014). A review of hyperacusis and future directions: part I. Definitions and manifestations. Am J Audiol.

[42] Vielsmeier V, Kreuzer PM, Haubner F, Steffens T, Semmler PR, Kleinjung T, Schlee W, Langguth B, Schecklmann M (2016). Speech comprehension difficulties in chronic tinnitus and its relation to hyperacusis. Front Aging Neurosci.

[43] Yang C, Jung J, Kim SH, Byun JY, Park MS, Yeo SG (2015). Comparison of clinical characteristics in patients with bilateral and unilateral tinnitus. Acta Oto Laryngol.

[44] Zeng FG (2013). An active loudness model suggesting tinnitus as increased central noise and hyperacusis as increased nonlinear gain. Hear Res.

